# Caraway yellows virus, a novel nepovirus from *Carum carvi*

**DOI:** 10.1186/s12985-019-1181-1

**Published:** 2019-05-27

**Authors:** Yahya Z. A. Gaafar, Katja R. Richert-Pöggeler, Angelika Sieg-Müller, Petra Lüddecke, Kerstin Herz, Jonas Hartrick, Christina Maaß, Roswitha Ulrich, Heiko Ziebell

**Affiliations:** 1 Julius Kühn Institute, Institute for Epidemiology and Pathogen Diagnostics, Messeweg 11-12, 38104 Braunschweig, Germany; 2 Regierungspräsidium Gießen, Schanzenfeldstrasse 8, 325578 Wetzlar, Germany

**Keywords:** Caraway, High throughput sequencing, Bipartite genome, Tubular structures, Nepovirus subgroup C

## Abstract

A novel nepovirus was identified and characterised from caraway, and tentatively named caraway yellows virus (CawYV). Tubular structures with isomeric virus particles typical for nepoviruses were observed in infected tissues by electron microscopy. The whole genome of CawYV was identified by high throughput sequencing (HTS). It consists of two segments with 8026 nt for RNA1 and 6405 nt for RNA2, excluding the poly(A) tails. CawYV-RNA1 shared closest nt identity to peach rosette mosaic virus (PRMV) with 63%, while RNA2 shared 41.5% with blueberry latent spherical virus (BLSV). The amino acid sequences of the CawYV protease-polymerase (Pro-Pol) and capsid protein (CP) regions share the highest identities with those of the subgroup C nepoviruses. The Pro-Pol region shared highest aa identity with PRMV (80.1%), while the CP region shared 39.6% to soybean latent spherical virus. Phylogenetic analysis of the CawYV-Pro-Pol and -CP aa sequences provided additional evidence of their association with nepoviruses subgroup C. Based on particle morphology, genomic organization and phylogenetic analyses, we propose CawYV as a novel species within the genus *Nepovirus* subgroup C.

**Electronic supplementary material:**

The online version of this article (10.1186/s12985-019-1181-1) contains supplementary material, which is available to authorized users.

## Main text

Viruses from the genus *Nepovirus* in the subfamily *Comovirinae* of the *Secoviridae* family possess a bipartite genome consisting of two positive ssRNAs with a 5′ viral protein genome-linked (VPg) and a 3′ poly(A) tail [1]. The RNA1 segment encodes the helicase, protease and its cofactor, replicase and the viral protein genome linked whereas the RNA2 segment encodes the movement and coat proteins [1, 2]. Nepoviruses are the only members of the family *Secoviridae* known to have a single CP [2]. Each of the two RNA segments are encapsidated separately in a non-enveloped icosahedral virion of 25–30 nm in diameter [1]. Nepoviruses can be transmitted non-persistently and non-circulatively by nematodes, mite and thrips [1, 3]. Seed and pollen transmissions are well-documented [1, 2]. In herbaceous plants, the symptoms induced are often transient with symptom recovery being a common outcome [2].

**Fig. 1:**
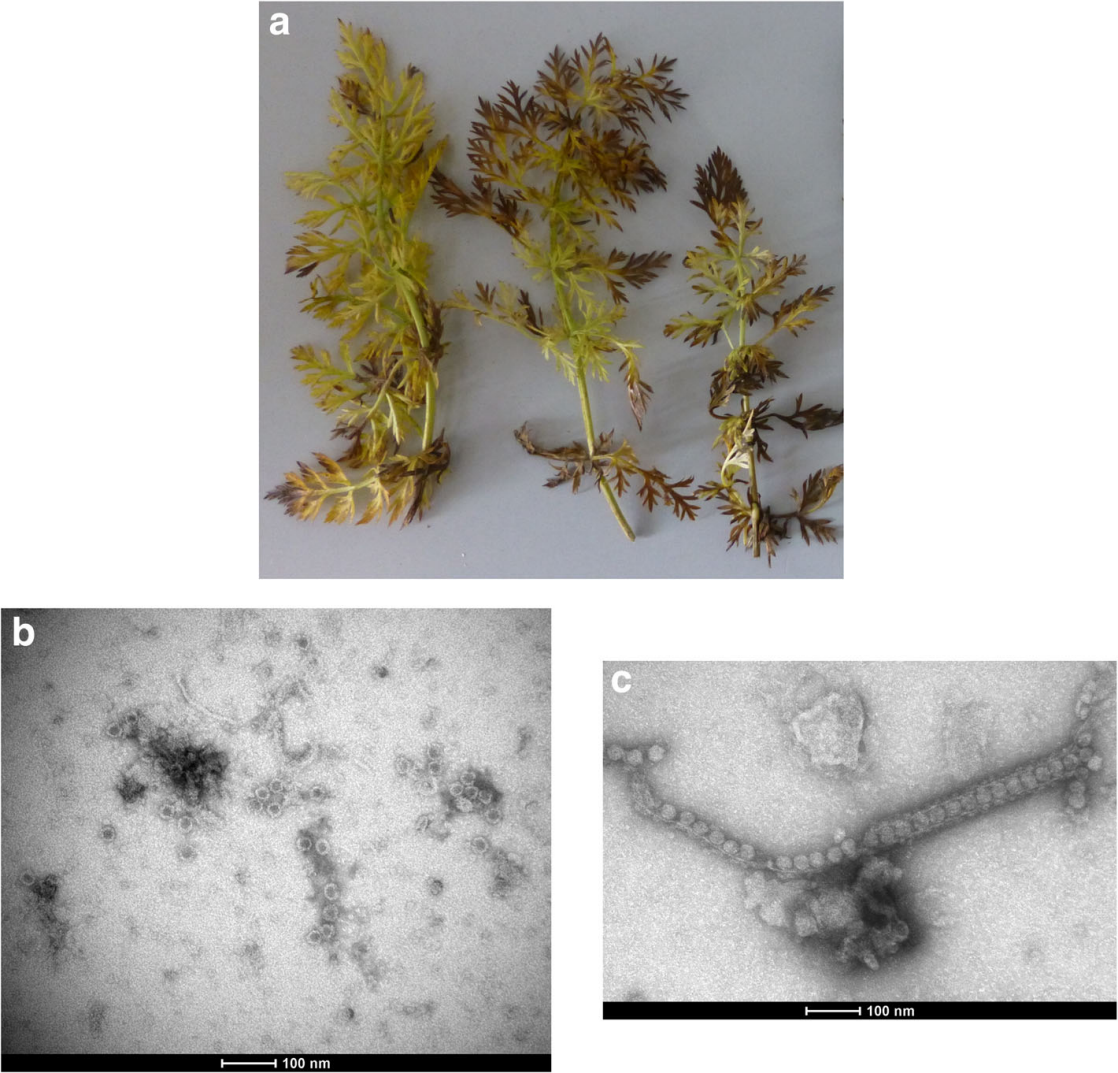
(**a**) Leaf symptoms of caraway yellows virus (CawYV) on caraway plants; (**b**) Electron micrograph of CawYV particles from the original infected caraway sample; (**c**) Electron micrograph showing tubular structure containing virus particles of CawYV in tissue homogenate of infected *Nicotiana benthamiana*

Caraway (*Carum carvi* L.) is an aromatic biennial plant in the *Apiaceae* family [4]. It is native to Europe, north Africa and western Asia [5, 6]. Caraway is used as a food flavour, fragrance additive, and for medical purposes as an antibacterial agent with antispasmodic, carminative, and appetite stimulant properties [4]. In 2016, an organic caraway field showed crop losses. A caraway plant sample with systemic yellowing was sent to Julius Kuehn-Institute (JKI) for analysis (Fig. 1a). The sample tested positive by DAS-ELISA using antiserum JKI 1283 developed against an uncharacterised nepovirus from carrot which is likely a strain of cherry leaf roll virus (CLRV) (unpublished data). The virus was mechanically transmitted to *Nicotiana benthamiana* and chlorotic local lesions were observed on inoculated leaves followed by systemic chlorosis and necrosis. Symptom recovery was not observed. The virus was provisionally named “caraway yellows virus” (CawYV).

Electron microscopy (EM) revealed the presence of isomeric virus particles of about 30 nm in diameter in preparations made from the original infected caraway sample (Fig. 1b), indicating the presence of a nepovirus. Additionally, tubules containing virus-like particles in tissue homogenate of *N. benthamiana* infected with the nepovirus were also observed by EM (Fig. 1c). This has also been shown for other nepoviruses e.g., grapevine fanleaf virus, where the movement and the capsid proteins act as components of tubular structures (required for cell to cell movement) that traverse the cell wall with the virus particles [1, 7].

To obtain the full genome of CawYV, double stranded RNA (dsRNA) was extracted from infected *N. benthamiana* using Double-RNA Viral dsRNA Extraction Mini Kit for Plant Tissue (iNtRON) following the manufacturer’s instructions. The extracted dsRNA was sent for library preparation and high throughput sequencing (HTS) at Eurofins GATC Biotech GmbH. The dsRNA was fragmented, strand specific cDNA was synthesized using random primers (the dsRNA was denatured at 99 °C for 2 min), followed by adapter ligation and adapter specific PCR amplification then sequencing on Illumina NovaSeq 6000 platform (2 × 150).

Using Geneious Prime (v. 2019.0.4), the raw reads (15,468,416) were quality trimmed, filtered, normalized, and error corrected followed by de novo assembly using Geneious assembler (Medium sensitivity/Fast setting). 36,634 contigs of lengths between 100 and 23,141 nt were generated. A BLASTn search of the contigs against a local database for viruses and viroids downloaded from NCBI showed that two contigs of 7180 and 6341 nt had 72% identity (73% coverage and zero E-value) to peach rosette mosaic virus (PRMV) and 79% (16% coverage and 1e-90 E-value) to blueberry latent spherical virus (BLSV), respectively. The 5′ ends of both RNA segments were confirmed using RNA ligase-mediated amplification of cDNA ends (RLM-RACE) [8]. The 3′ ends of the two RNA segments were determined by using an oligo(d)T primer for cDNA synthesis followed by PCR using virus specific primers and the oligo(d)T primer. The primers used for the 5′ and 3′ ends confirmation are listed in Additional file 1: Table S1. The PCR products were cloned, sequenced and the resulting sequences were assembled using the map to reference tool and the original assembled contigs as references. 72,977 of the quality trimmed reads were assembled to CawYV complete genome. The assembled genome of CawYV was 8026 nt for RNA segment 1 and 6405 nt for RNA segment 2 (excluding poly(A) tails). The sequences were deposited in the GenBank database under accessions MK492273 and MK492274. For diagnostic purposes and to confirm the presence of CawYV in symptomatic leaf tissue, a primer pair was designed using Primer 3 tool in Geneious (HZ-636 5′ TGA AGA TCC GGG AAA GGC AC 3′ and HZ-637 5′ ACG CTT TCC ACT CTC ACC TG 3′) [9]. The presence of CawYV was confirmed in the infected plants by RT-PCR using OneTaq One-Step RT-PCR Kit (NEB) resulting in amplicons of 481 bp (data not shown).

**Fig. 2:**
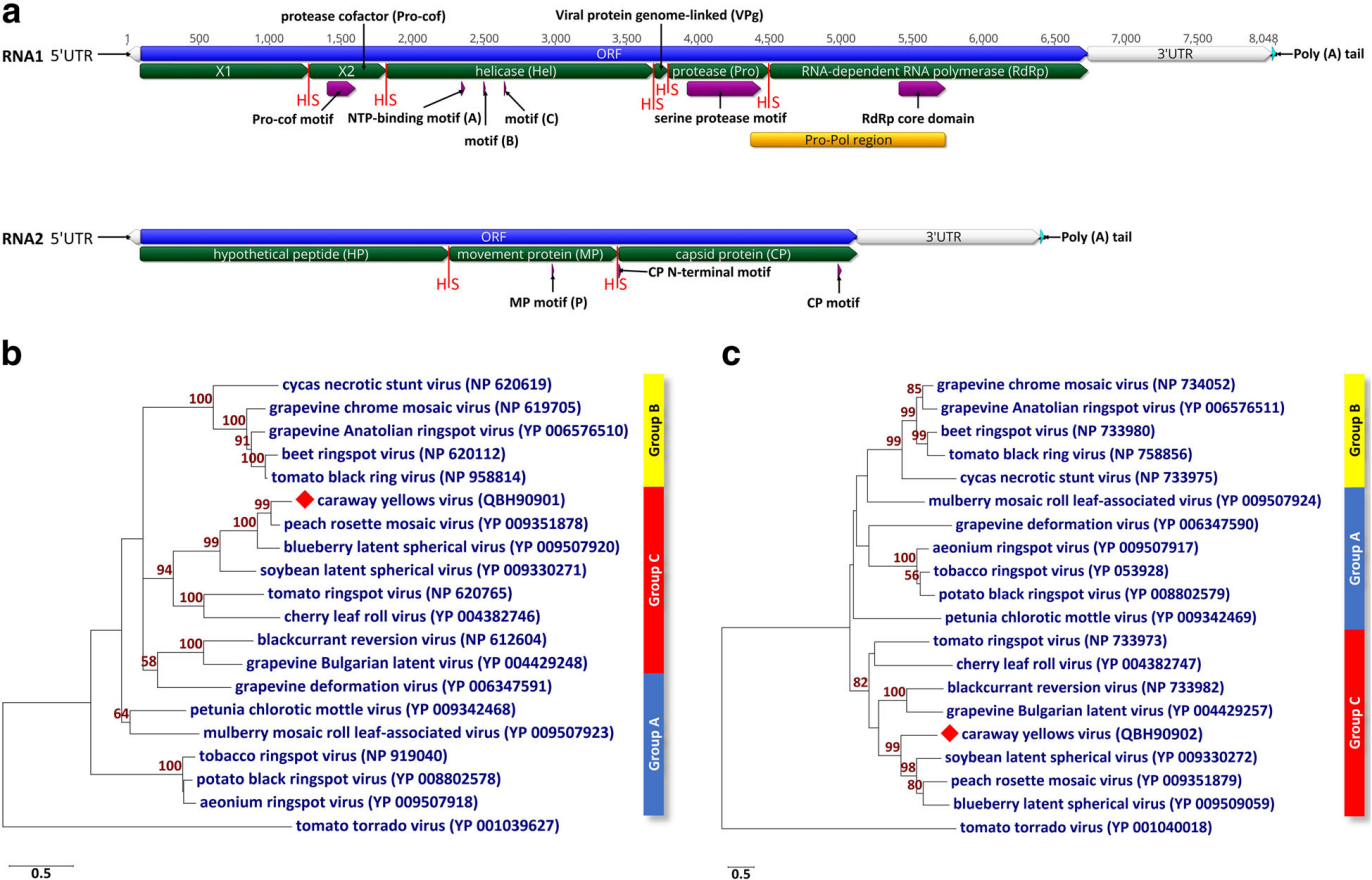
(**a**) Genome organization of CawYV-RNA1 and -RNA2. Each of RNAs 1 and 2 contain a single large open reading frame (in blue). The predicted putative peptides are shown in green, separated by the predicted cleavage site (H/S) (red). The RNA1 polyprotein contains X1 protein, protease cofactor (Pro-cof/X2), helicase (Hel), viral protein genome-linked (VPg), protease (Pro) and RNA-dependent RNA polymerase (RdRP). RNA2 encodes for a polyprotein with hypothetical protein (HP), movement protein (MP) and capsid protein (CP). The conserved nepovirus sequences (domains and motifs) are shown in violet. The protease-polymerase (Pro-Pol) region of CawYV-RNA1 starts with the serine (S) of the protease motif and ends with the (GDD) motif of the polymerase (shown in gold). (**b**) Maximum-likelihood (ML) phylogenetic trees showing the relationships between CawYV and members of the genus *Nepovirus* based on aa alignments of the Pro-Pol region and (**c**) the capsid protein (CP) region. Numbers on branches indicate the bootstrap percentages (1000 replicates, ≥50% are shown). Tomato torrado virus (genus *Torradovirus*, family *Secoviridae*) is an outgroup

Further analyses of the CawYV sequence confirmed its identity as a nepovirus. In analogy to other nepoviruses, CawYV RNA1 contains an open reading frame (nt position 92 to 6733) encoding a polyprotein of 2213 aa in length. Pairwise comparisons of nt and aa sequences of this ORF to its homologues of the other nepoviruses were performed using ClustalW [10]. The results show that the highest similarity was shared with PRMV at 66.1% on nt and 68.1% aa levels, respectively (Table 1). By searching for the different nepovirus motifs using the motif searching tool in Geneious, the locations of the putative protease cofactor (Pro-cof), the NTP-binding helicase domain (Hel), the serine protease domain (Pro), and the RNA-dependent RNA polymerase (RdRp) core domain were found in the RNA1-encoded polyprotein [11]. The putative viral protease cofactor motif (FX_27_WX_11_LX_21_LXE) was located at aa residues 438–502. The typical NTP-binding helicase motif A (GX_4_GKS), motif B (D), and motif C (N) were found at aa 752–759, 803, and 852, respectively. A serine protease motif was found at aa 1280–1449 (HX_40_EX_106_**S**GX_8_GX_5_GXHX_2_G) of the CawYV RNA1 polyprotein sequence (Fig. 2a). The serine at this position is unusual for members of the *Picornavirales* (where cysteine is usually encoded) but was described for some members of genus *Nepovirus* subgroup C i.e., BLSV, CLRV, PRMV and soybean latent spherical virus (SLSV). The RNA-dependent RNA polymerase (RdRp) core domain was located at aa 1774–1880 (DX_4_DX_56_GX_3_TX_3_NX_33_GDD). Pairwise analysis of the protease-polymerase (Pro-Pol) region aa sequences showed a closest identity to PRMV Pro-Pol with 80.1% (Table 1).

**Table 1:** Characteristics and pairwise nucleotide (nt) and amino acid (aa) alignments of the different regions of caraway yellows virus (CawYV) and selected members of subgroup C of the genus *Nepovirus* i.e., blueberry latent spherical virus (BLSV), blackcurrant reversion virus (BCRV), cherry leaf roll virus (CLRV), grapevine Bulgarian latent virus (GBLV), peach rosette mosaic virus (PRMV), soybean latent spherical virus (SLSV) and tomato ringspot virus (ToRSV)

Virus			CawYV	PRMV	BLSV	SLSV	BCRV	GBLV	ToRSV	CLRV
nt length	RNA 1	Accession no.	MK492273	NC_034214	NC_038764	NC_032270	NC_003509	NC_015492	NC_003840	NC_015414
		Complete /−poly(A)	8026	8014	7960	8170	7711	7452	8214	7918
		5′UTR	91	41	61	13	66	87	77	11
		ORF	6642	6504	6519	6588	6285	6288	6594	6339
		3′UTR	1293	1469	1380	1569	1360	1077	1543	1568
	RNA 2	Accession no.	MK492274	NC_034215	NC_038763	NC_032271	NC_003502	NC_015493	NC_003839	NC_015415
		Complete /−poly(A)	6405	5956	6344	5776	6405	5821	7271	6360
		5′UTR	94	47	55	23	161	189	75	11
		ORF	5022	4425	4896	4197	4881	4500	5649	4770
		3′UTR	1289	1484	1393	1556	1363	1132	1547	1579
Pairwise identity %										
nt %	RNA 1	Complete /−poly(A)		63	60.8	49.6	38.5	37	36	33.8
		5′UTR		56.1	55.7	30.8	40.9	31.6	52	63.6
		ORF		66.1	62.4	53.5	40.4	39.2	36.7	36.3
		3′UTR		51.4	53.8	34	40.5	36.5	27	30.9
	RNA 2	Complete /−poly(A)		41.3	41.5	37.7	35.9	30.9	38.6	35.6
		5′UTR		37.2	30.9	34.8	35.9	36.2	41.3	63.6
		ORF		39.9	38.5	39	37	31.8	40.9	37.7
		3′UTR		51	55.2	33.4	36.1	37.1	29.4	30.6
aa %	RNA 1	ORF		68.1	62.5	48.1	24.5	22.8	23.1	22.6
		X1		27.9	29.7	22.9	12.2	10.6	10.8	10.8
		X2 Pro-cof		52.9	53.2	33.9	25.8	21.4	15.6	18.5
		Hel		82.5	75.6	59.3	28	24.1	22.6	23.2
		VPg		75	57.6	56.2	6.1	27.3	25.9	37.9
		Pro		79	66.8	51.3	27.6	23.5	24.7	23.1
		RdRp		75.5	68.6	53.9	33.8	33.6	33.7	30.9
		Pro-Pol		80.1	70.2	54.9	7.1	35.3	36.5	34.6
	RNA 2	ORF		22.9	20.1	19	13.1	12.2	22.3	19
		HP		23.3	13.1	6.5	7.3	7.6	8.2	7
		MP		10	8.4	7.5	8.5	4.1	52.2	54.6
		CP		36.5	34.7	39.6	24.3	24.8	26.1	20

CawYV-RNA2 contains an open reading frame (nt position 95 to 5116) encoding a polyprotein of 1673 aa in length. Pairwise comparisons of RNA2-ORF nt and aa sequences to the homologues of other nepoviruses showed the highest similarity with PRMV with 31% nt and 21.3% aa identities (Table 1). The conserved movement protein motif (P) was found at aa position 962 [12]. The CP N-terminal five amino acid residues (SGLEE) together with an alternate capsid protein (CP) motif (FXFYGWS) were located at aa positions 1119–1122 and 1631–1637 [11, 13]. Pairwise analysis of the CP region showed that it shares highest aa identity to SLSV (39.6%, Table 1).

Each of the CawYV polypeptides is predicted to be proteolytically cleaved into putative peptides by the virus-encoded protease. Sequence alignment of all nepovirus ORF aa sequences suggest a putative proteolytic cleavage sites at dipeptides (H/S). This potential cleavage site was not identified before in the *Secoviridae* members. The conserved histidine in the substrate-binding pocket of the protease is known for members of the subgroup C, however the known cleavage sites are Q/G, Q/S or D/S (confirmed experimentally) [1]. The H/S dipeptide is also found in SLSV, BLSV and PRMV. Although the VPg motif was not confirmed in the polyprotein of RNA1, the location of the putative VPg domain could be determined by the H/S dipeptides between the NTP-binding helicase and the protease using sequence alignment (Fig. 2a). Additionally, the X1 putative protein was identified at the N terminus of RNA1 polyprotein by the presence of a H/S dipeptide potential cleavage site before the protease cofactor motif of X2 (Fig. 2a). The 5′ untranslated regions (UTR) of the two RNAs were 91 nt for RNA1 and 94 nt for RNA2 and shared 61.3% nt identity to each other. The 3’UTRs (1293 and 1289 nt for RNA 1 and 2 respectively, excluding the poly(A) tail) are 98.4% identical.

A maximum likelihood tree using MEGA7 software (v 7.0.26) based on the aa alignments of the Pro-Pol and the CP regions were additional evidence for the relatedness of CawYV to the *Nepovirus* subgroup C (Fig. 2b and c) [14].

The International Committee on Taxonomy of Viruses (ICTV) suggests the following criteria for species demarcation [1]: distinct host range; distinct vector specificity; absence of cross-protection; differences in antigenic reactions; absence of reassortment between RNA1 and RNA2; Pro-Pol region aa < 80% and CP region aa < 75% identities. Although the host range was not studied, the closest relatives of CawYV, i.e., PRMV and BLSV, are not known to infect members of the *Apiaceae* family. The serological cross-reactivity is well known for members of the same genus in the family *Secoviridae* [1]. This might explain why our antiserum raised against an uncharacterised strain of CLRV reacted with CawYV. Further investigations are necessary to test the antiserum against other nepoviruses, and attempts are currently underway to develop a CawYV-specific antiserum. When compared to the closest relative PRMV, the Pro-Pol region of CawYV is slightly above the species demarcation limit by 0.1%. However, this was also observed for other nepoviruses e.g., beet ringspot virus (BRSV) and tomato black ring virus (TBRV) that share 89% aa identity at the Pro-Pol [1] but are yet classified as distinct species. However, the caraway virus-CP region is very different to other nepoviruses sharing only 39.6% aa identity with SLSV. Based on these results, we propose the assignment of CawYV as a new virus species within the subgroup C of the genus *Nepovirus*. Further studies are needed to investigate the natural mode of transmission and the biological characteristics of CawYV.

## Additional file

Additional file 1: **Table S1.** List of the primers used for caraway yellows virus 5′ and 3′ ends confirmation. (DOCX 18 kb)

## Data Availability

The datasets used and/or analysed during the current study are available from the corresponding author on reasonable request.

## Abbreviations

aa: amino acid
BCRV: blackcurrant reversion virus
BLSV: blueberry latent spherical virus
BRSV: beet ringspot virus
CawYV: caraway yellows virus
CLRV: cherry leaf roll virus
CP: capsid protein
DAS-ELISA: double antibody sandwich enzyme-linked immunosorbent assay
dsRNA: double stranded RNA
EM: electron microscopy
GBLV: grapevine Bulgarian latent virus
Hel: helicase
HTS: high throughput sequencing
ICTV: International Committee on Taxonomy of Viruses
JKI: Julius Kuehn-Institute
kDa: kilo Dalton
nm: nanometer
nt: nucleotide
PRMV: peach rosette mosaic virus
Pro: protease
Pro-cof: protease cofactor
Pro-Pol: protease-polymerase
RdRp: RNA-dependent RNA polymerase
RLM-RACE: RNA ligase-mediated amplification of cDNA ends
RT-PCR: reverse transcription polymerase chain reaction
SLSV: soybean latent spherical virus
TBRV: tomato black ring virus
ToRSV: tomato ringspot virus
UTR: untranslated regions
VPg: protein genome-linked
