# Supplementary material for: Caraway yellows virus, a novel nepovirus from Carum carvi
Source: Virol J. 2019 May 27;16:70. doi: 10.1186/s12985-019-1181-1 (PMC6537451; doi:10.1186/s12985-019-1181-1)
Supplement: Supplementary file 1 — Table S1. List of the primers used for caraway yellows virus 5′ and 3′ ends confirmation. (DOCX 18 kb) [file 12985_2019_1181_MOESM1_ESM.docx]

Additional file 1: **Table S1:** List of the primers used for caraway yellows virus 5’ and 3’ ends confirmation.

| **Location** | **Virus Specific Primers** | | |
| --- | --- | --- | --- |
|  | **Name** | **Sequence** | **nt position** |
| **RNA1-5’ end** | **HZ-648** | **5’ GCT TGT TTA GTA GCG GCT GC 3’** | **504-485** |
|  | **HZ-649** | **5‘ GCA ATC TGC AAA TAT CGT GGC T 3‘** | **324-303** |
| **RNA2-5’ end** | **HZ-644** | **5’ CAA TGC CCA CAA GCT TAG CG 3’** | **464-445** |
|  | **HZ-645** | **5’ ACT TTG TCA TAG CGC TCG GC 3’** | **321-302** |
| **Adaptor** | **HZ-481** | **5’ PO4-GAT CCA CTA GTT CTA GAG CGG C-AminoC3 3’** | **NA** |
| **Adaptor complement** | **HZ-482** | **5’ GCC GCT CTA GAA CTA GTG GAT C 3’** | **NA** |
| **RNA1-3’ end** | **HZ-670** | **5’ GGG AGA CAT AGC ACC TCT TCT 3’** | **6625-6645** |
|  | **HZ-671** | **5’ GAC ATG TCT CCA GAC CTA TTT TCT 3’** | **6666-6689** |
| **RNA2-3’ end** | **HZ-672** | **5’ ACC CCA GCA GCT TTC ACT AC 3’** | **5024-5043** |
|  | **HZ-673** | **5’ CTA AGC CGA GAG AGG AAC GC 3’** | **5073-5092** |
| **Poly(T)18** | **HZ-012** | **5’ CCT CGG GCA GTC CTT TTT TTT TTT TTT TTT T 3’** | **NA** |

NA: not applicable.
